# Supplementary material for: Establishment and validation of a novel CD8+ T cell-associated prognostic signature for predicting clinical outcomes and immunotherapy response in hepatocellular carcinoma via integrating single-cell RNA-seq and bulk RNA-seq
Source: Discov Oncol. 2024 Jun 20;15:235. doi: 10.1007/s12672-024-01092-z (PMC11190115; doi:10.1007/s12672-024-01092-z)
Supplement: Supplementary file 1 — Supplementary file1 (DOCX 13487 KB) Supplementary Table 1: The primer sequence of genes. Supplementary Fig. 1: The work flowchart of the study. Abbreviations: WGCNA: Weighted correlation network analysis, ICGC: International Cancer Genome Consortium, TME: tumor microenvironment, TIICs: Tumor-infiltrating immune cells. Supplementary Fig. 2: Integration and clustering of scRNA-Seq. A Annotation and visualization of cell subsets. B A total of 15 distinct clusters were identified via the t-SNE and UMAP algorithms. C Heatmap of cluster markers expression in each cluster. Supplementary Fig. 3: A GO enrichment analysis for CD8 T cell-related genes, including biological process (BP), cellular component (CC), and molecular function (MF). B KEGG enrichment analysis for CD8 T cell-related genes. Supplementary Fig. 4: The predictive value of the risk score in the ICGC external cohort model. A-B Univariate and multivariate Cox analysis of risk scores. C A nomogram was constructed based on risk scores. D The calibration curve of the nomogram. E The ROC curve of the nomogram. F The ROC curves of risk score. G The C-index of risk score. Supplementary Fig. 5: The KEGG signaling pathways enriched by the risk score. A High-risk score group. B Low-risk score group. Supplementary Fig. 6: Expression of risk genes in T cell clusters. A-B The violin map and bubble plot show the expression of risk genes in T cell clusters. C The t-SNE plot demonstrates the risk gene expression levels in T-cell clusters. Supplementary Fig. 7: IHC staining images of risk genes in HCC tissues and normal liver tissues were obtained from the HPA database. A IKBKE. B ATP1B3. C ADA. D BATF. [file 12672_2024_1092_MOESM1_ESM.docx]

**Supplemental Table**

Table S1: The primer sequence of genes.

| Gene name | Primer sequence |
| --- | --- |
| GAPDH-forward | GGAGCGAGATCCCTCCAAAAT |
| GAPDH-reverse | GGCTGTTGTCATACTTCTCATGG |
| IKBKE-forward | TGCCTGAGGATGAGTTCCTG |
| IKBKE-reverse | CGATGCACAATGCCGTTCT |
| ATP1B3-forward | AACCCGACCACCGGAGAAT |
| ATP1B3-reverse | TGAGAGTCTGAAGCATAACCCA |
| MSC-forward | CCCCGACACTAAGCTCTCCA |
| MSC-reverse | GTAGCCGTTCTCATAGCGGT |
| ADA-forward | GCCTTCGACAAGCCCAAAGTA |
| ADA-reverse | CTCTGCTGTGTTAGCTGGGAG |
| BATF-forward | TATTGCCGCCCAGAAGAGC |
| BATF-reverse | GCTTGATCTCCTTGCGTAGAG |

**Supplementary Figure**

**
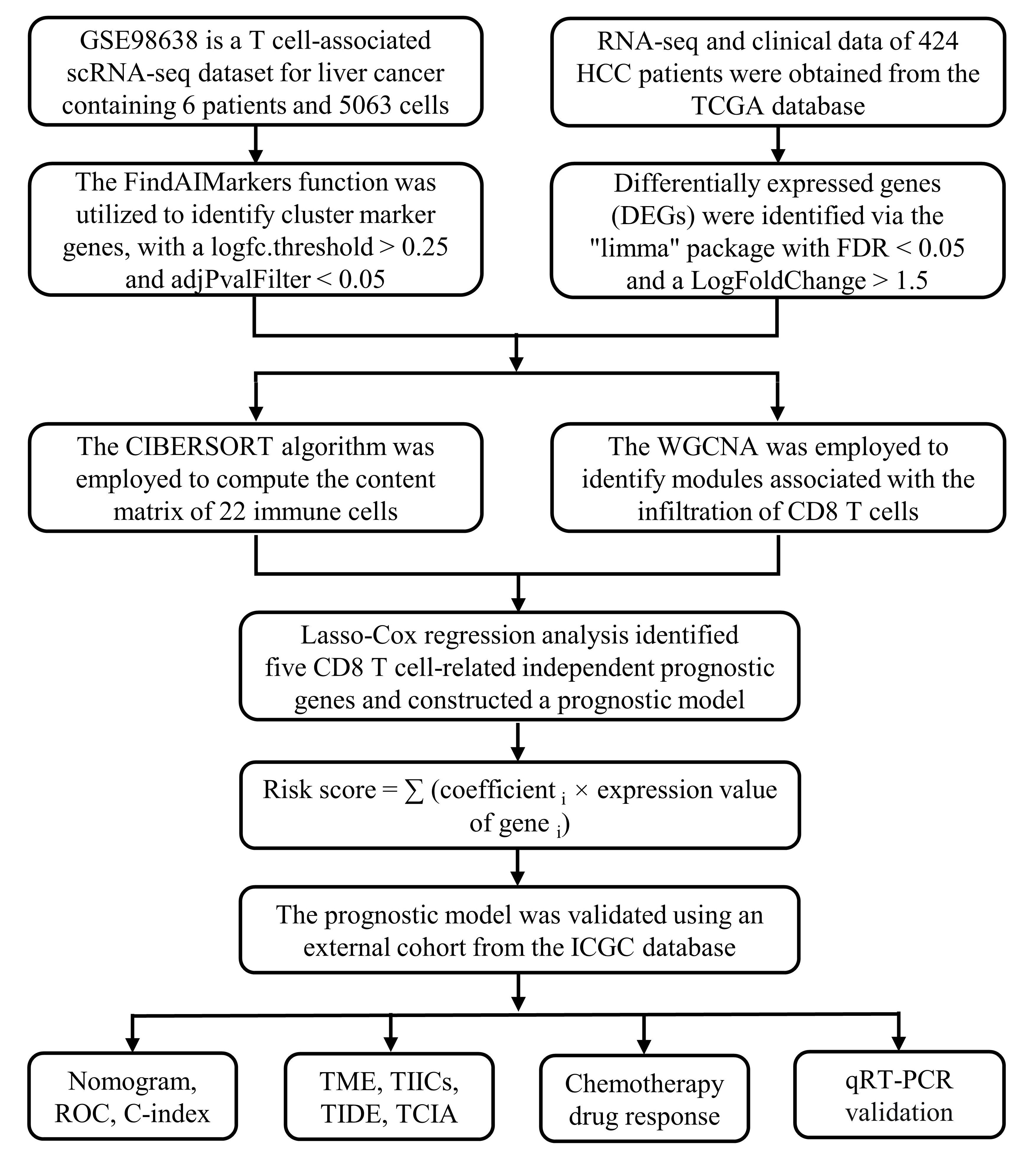
**

**Supplementary Figure 1:** The work flowchart of the study. Abbreviations: WGCNA: Weighted correlation network analysis, ICGC: International Cancer Genome Consortium, TME: tumor microenvironment, TIICs: Tumor-infiltrating immune cells.


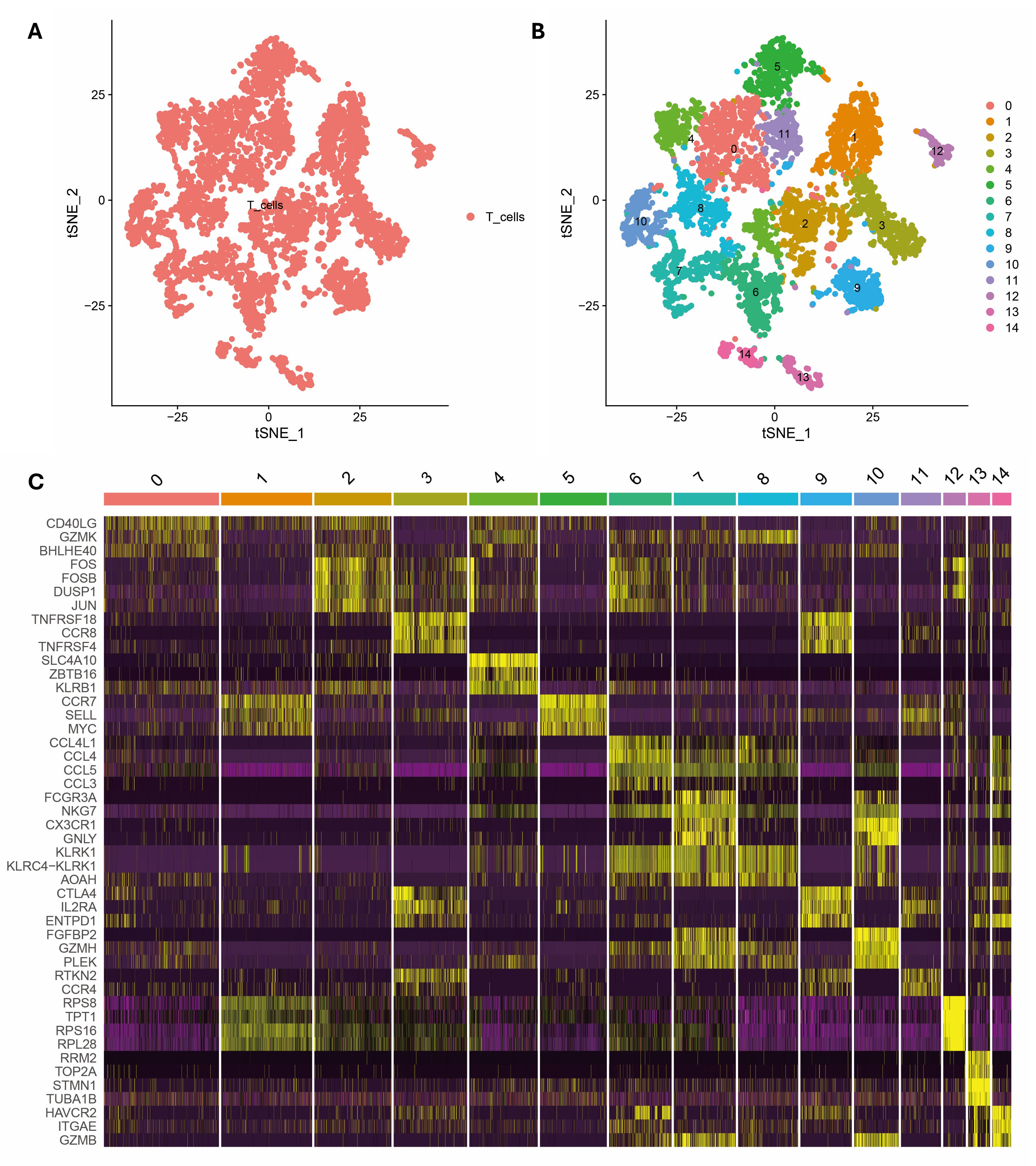


**Supplementary Figure 2:** Integration and clustering of scRNA-Seq. **A** Annotation and visualization of cell subsets. **B** A total of 15 distinct clusters were identified via the t-SNE and UMAP algorithms. **C** Heatmap of cluster markers expression in each cluster.

**
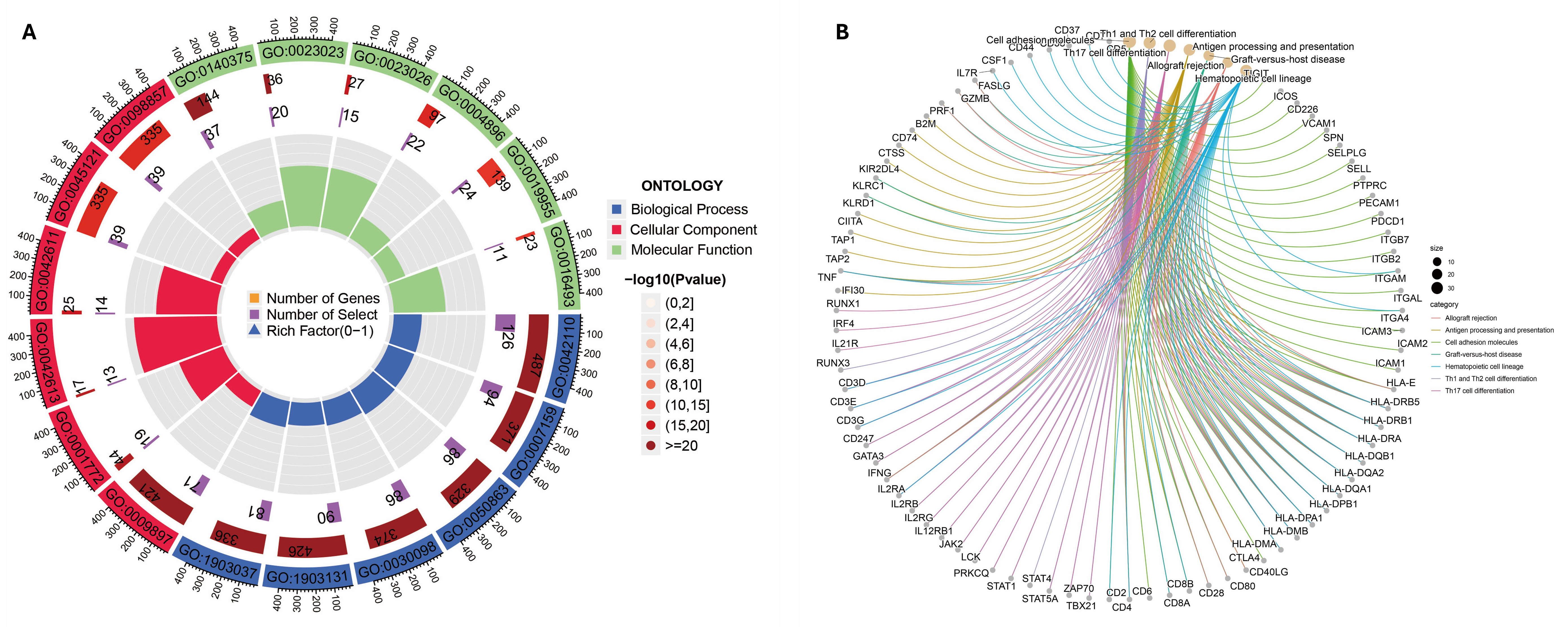
**

**Supplementary Figure 3: A** GO enrichment analysis for CD8 T cell-related genes, including biological process (BP), cellular component (CC), and molecular function (MF). **B** KEGG enrichment analysis for CD8 T cell-related genes.

**
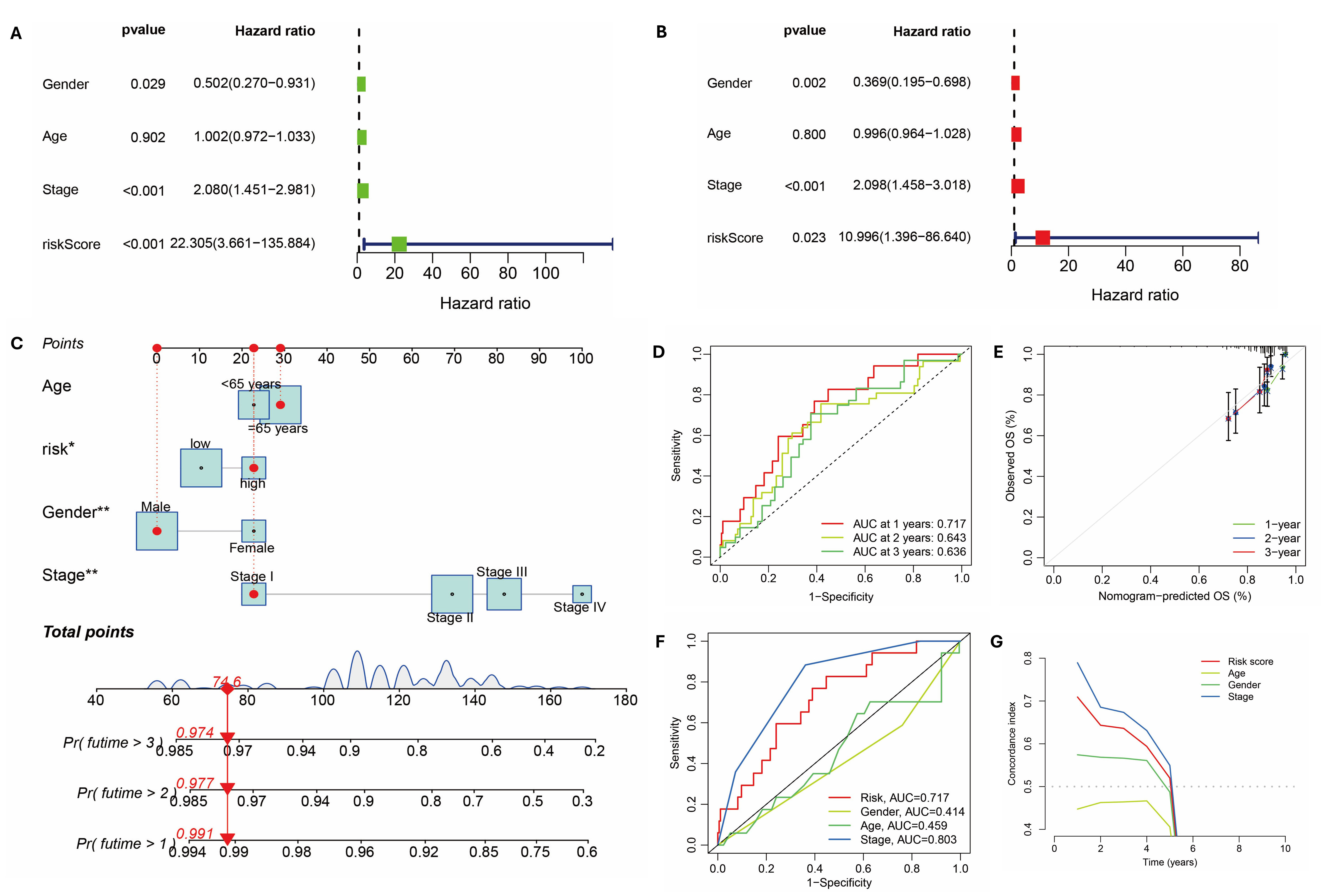
**

**Supplementary Figure 4:** The predictive value of the risk score in the ICGC external cohort model. **A-B** Univariate and multivariate Cox analysis of risk scores. **C** A nomogram was constructed based on risk scores. **D** The calibration curve of the nomogram. **E** The ROC curve of the nomogram. **F** The ROC curves of risk score. **G** The C-index of risk score.

**
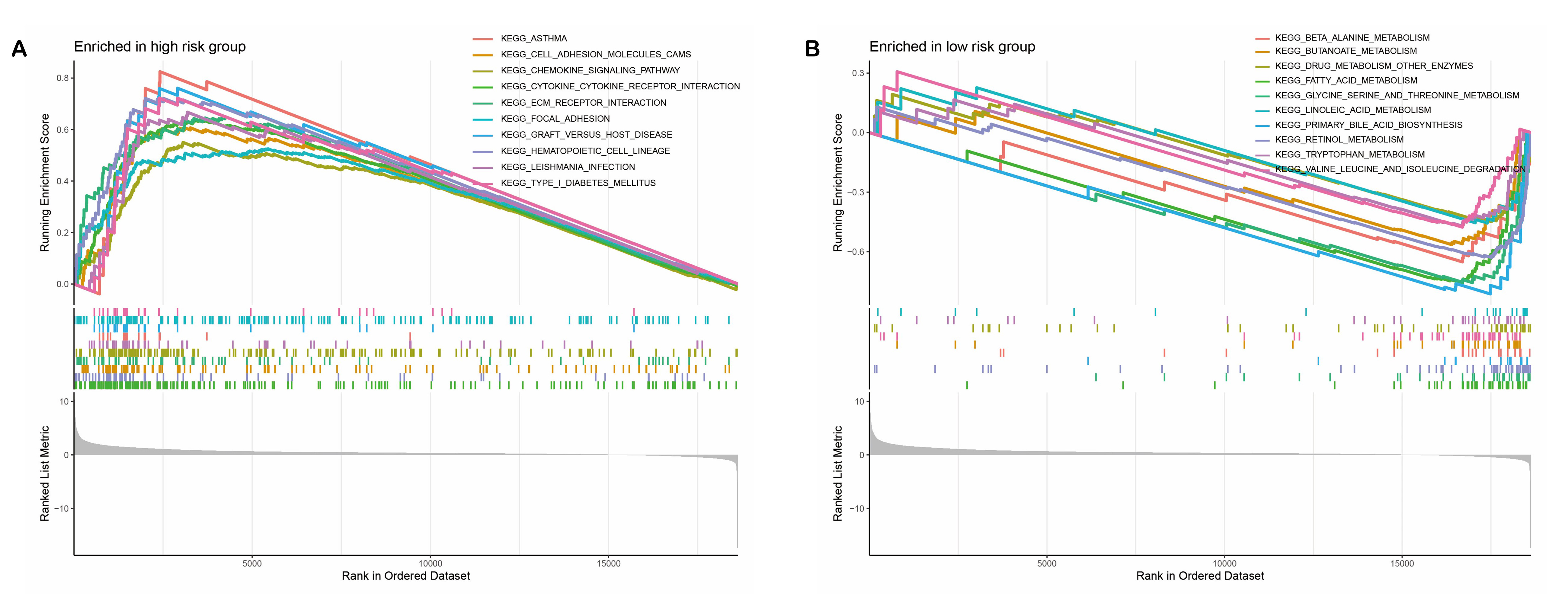
**

**Supplementary Figure 5:** The KEGG signaling pathways enriched by the risk score. **A** High-risk score group. **B** Low-risk score group.


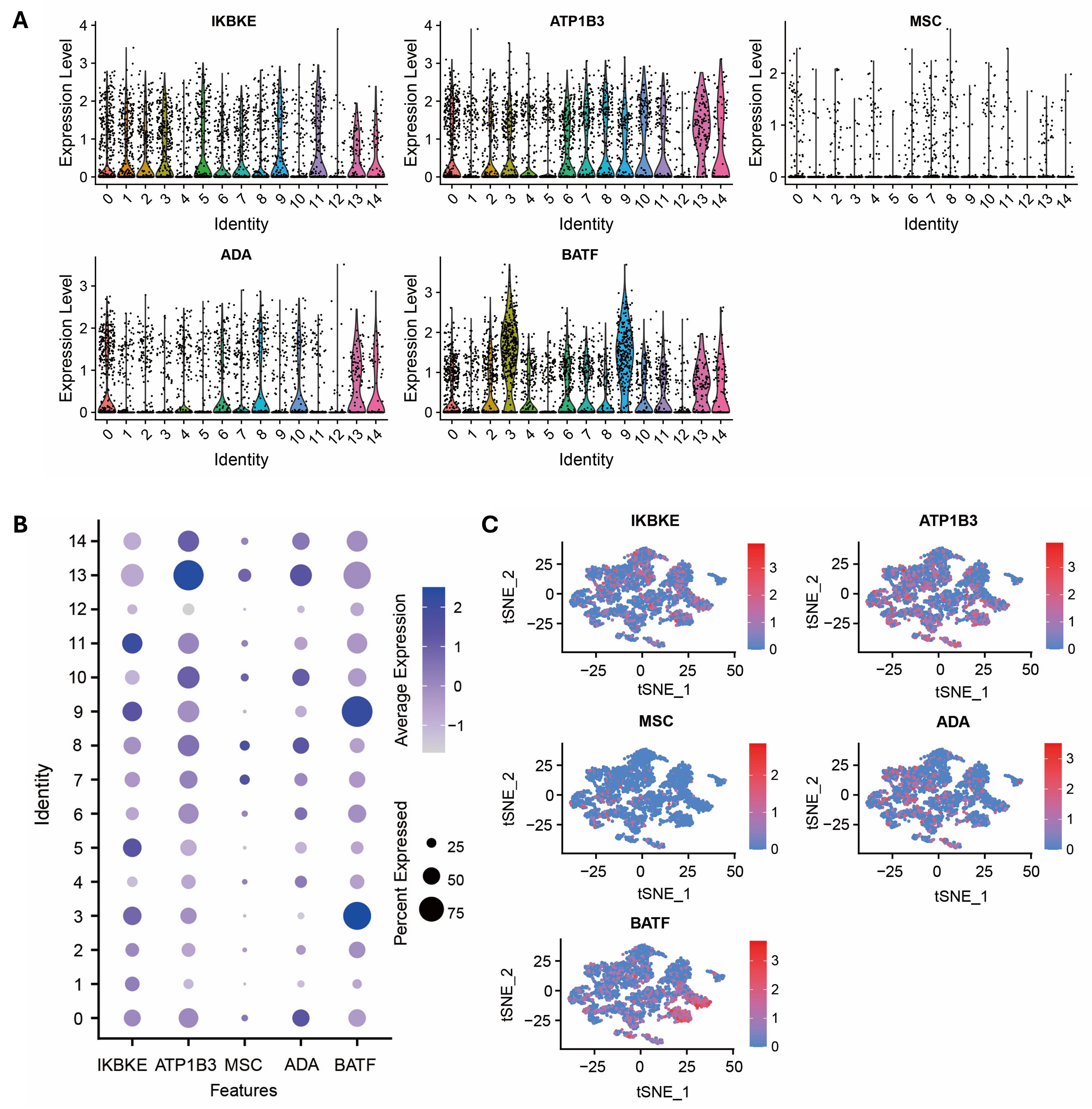


**Supplementary Figure 6:** Expression of risk genes in T cell clusters. **A-B** The violin map and bubble plot show the expression of risk genes in T cell clusters. **C** The t-SNE plot demonstrates the risk gene expression levels in T-cell clusters.

**
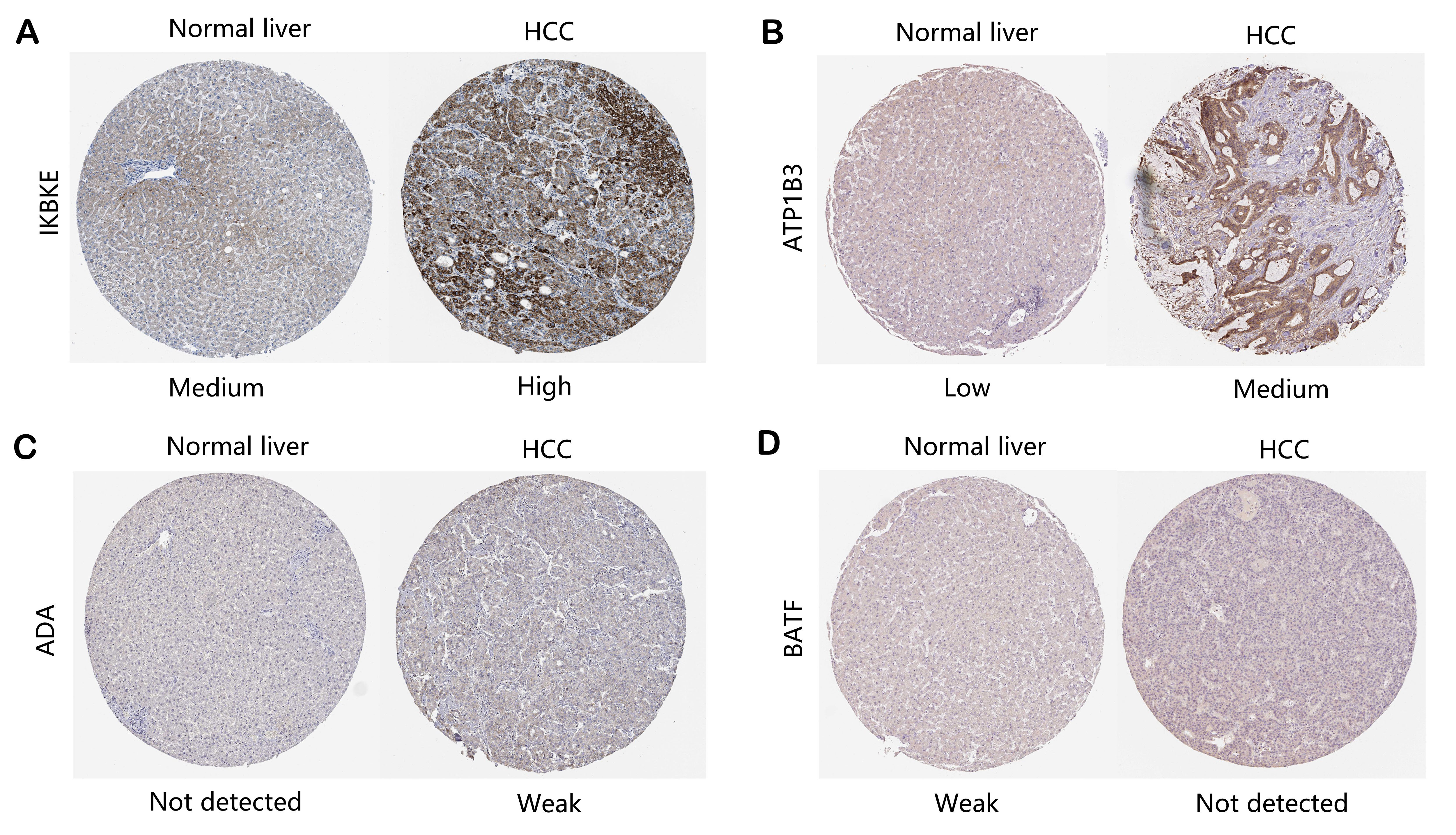
**

**Supplementary Figure 7:** IHC staining images of risk genes in HCC tissues and normal liver tissues were obtained from the HPA database. **A** IKBKE. **B** ATP1B3. **C** ADA. **D** BATF.
